# Don’t You Forget About Me: Gendered Associations of Dyadic Cognition and Loneliness in SHARE

**DOI:** 10.1093/geroni/igaf122.1938

**Published:** 2025-12-31

**Authors:** Jeffrey Stokes, Heather Farmer, Lisa Jessee, Martina Luchetti, Angelina R Sutin

**Affiliations:** University of Massachusetts Boston, Boston, Massachusetts, United States; University of Delaware, Newark, Delaware, United States; University of Cologne, Cologne, Nordrhein-Westfalen, Germany; Florida State University College of Medicine, Tallahassee, Florida, United States; Florida State University College of Medicine, Tallahassee, Florida, United States

## Abstract

Recent research has sought to contextualize the experience of loneliness within social networks as well as within intimate couples. Loneliness is influenced not only by the number but also by the quality of individuals’ close relationships, and it can have health consequences for intimate partners, as well, including their cognitive functioning. However, little attention has been paid to potential reciprocal effects of cognition on loneliness. That is, does having a partner experiencing cognitive decline lead to greater loneliness over time as well? We use longitudinal dyadic data from Waves 6 (2015) and 9 (2022) of the Survey of Health, Ageing, and Retirement in Europe (SHARE; N = 16,392) to examine whether individuals’ own and/or their partners’ cognitive functioning at baseline – measured using verbal fluency and episodic memory – are associated with changes in loneliness over the 7-year period. Moreover, we examined potential variation in effects by gender and age. Multilevel longitudinal dyadic lagged dependent variable regression models revealed that (a) poorer verbal fluency and episodic memory at baseline were associated with increased loneliness over time; (b) a partner’s poorer verbal fluency at baseline was associated with increased loneliness over time for women, but not for men; and (c) a partner’s poorer memory at baseline was associated with increased loneliness among those 70 and older, but not among younger respondents; this effect was strengthened at older ages. We discuss the implications of these findings for theory and future research concerning gender and relationships, cognition, and loneliness within aging couples.

